# Genetic heritability as a tool to evaluate the precision of 24-hour recall dietary questionnaire variables in UK Biobank

**DOI:** 10.3389/fgene.2022.1070511

**Published:** 2023-01-04

**Authors:** Joanne B. Cole, Kenneth E. Westerman, Alisa K. Manning, Jose C. Florez, Joel N. Hirschhorn

**Affiliations:** ^1^ Programs in Metabolism and Medical and Population Genetics, The Broad Institute of MIT and Harvard, Cambridge, MA, United States; ^2^ Diabetes Unit and Center for Genomic Medicine, Massachusetts General Hospital, Boston, MA, United States; ^3^ Department of Medicine, Harvard Medical School, Boston, MA, United States; ^4^ Division of Endocrinology, Boston Children’s Hospital, Boston, MA, United States; ^5^ Department of Biomedical Informatics, University of Colorado School of Medicine, Aurora, CO, United States; ^6^ Clinical and Translational Epidemiology Unit, Mongan Institute, Massachusetts General Hospital, Boston, MA, United States; ^7^ Department of Genetics, Harvard Medical School, Boston, MA, United States

**Keywords:** heritability, nutrigenomics, nutritional epidemiology, 24-hour diet recall questionnaires, relative validity, phenotyping, empirical bayes, longitudinal data

## Abstract

A variety of statistical approaches in nutritional epidemiology have been developed to enhance the precision of dietary variables derived from longitudinal questionnaires. Correlation with biomarkers is often used to assess the relative validity of these different approaches, however, validated biomarkers do not always exist and are costly and laborious to collect. We present a novel high-throughput approach which utilizes the modest but importantly non-zero influence of genetic variation on variation in dietary intake to compare different statistical transformations of dietary variables. Specifically, we compare the heritability of crude averages with Empirical Bayes weighted averages for 302 correlated dietary variables from multiple 24-hour recall questionnaires in 177 K individuals in UK Biobank. Overall, the crude averages for frequency of consumption are more heritable than their Empirical Bayes counterparts only when the reliability of that item across questionnaires is high (measured by intra-class correlation), otherwise, the Empirical Bayes approach (for both unreliably measured frequencies and for average quantities independent of reliability) leads to higher heritability estimates. We also find that the more heritable versions of each dietary variable lead to stronger underlying statistical associations with specific genetic loci, many of which have well-known mechanisms, further supporting heritability as an alternative metric for relative validity in nutritional epidemiology and beyond.

## Introduction

Dietary data are commonly collected longitudinally to enhance precision of dietary intake estimates. A variety of statistical approaches have been developed to best use this type of data in nutritional epidemiology. The simplest univariate approach is to collapse the data points per individual into a single aggregate mean or median, most appropriate when the variable is expected to be stable over time (Schober and Vetter, 2018). However, usual dietary intake is often estimated from unstable dietary questionnaire data with high day-to-day variation, such as from the 24-hour recall (24HR) questionnaire which records all foods and beverages consumed in a single day. For foods and beverages not consumed on a daily basis, the simple average approach over a small number of days is typically not adequate for capturing true habitual intake (Dodd et al., 2006). Nutritional epidemiologists and statisticians have developed methods to best handle this specific problem, most often applying sophisticated methods that estimate the true distribution of usual intake after accounting for within-person variability or for the correlation between observations in a mixed effects model (Dodd et al., 2006; Tooze et al., 2006). Extensions of these approaches apply the regression calibration approach for measurement error correction to estimate individual usual intake as the estimated conditional expectation given the empirically observed 24HR data [i.e., the Empirical Bayes (EB) method], which then allows for downstream association with an outcome of interest (Kipnis et al., 2009).

A key outstanding challenge addresses how best to evaluate and compare the performance of these various methods in increasing phenotype precision. The most common approach in epidemiology to assess relative validity is to demonstrate an improvement in the correlation of the processed phenotype with a “gold standard” measurement. The correlation of total energy intake or protein intake with doubly-labeled water (International Atomic Energy Agency, 1990) or urine protein levels (Greenwood et al., 2019), respectively, are classic examples of evaluating the validity of dietary intake derived from diet questionnaires. The EB method for estimating individual usual intake along with the incorporation of key covariates has also demonstrated improved phenotype precision when specifically testing the association between fish intake and blood mercury levels (Kipnis et al., 2009). In principle, the strength of association becomes stronger when noise and measurement error is reduced (Paeratakul et al., 1998; Willett, 2012). However, these approaches are only viable when a known gold standard measurement of the outcome of interest exists; these gold-standard methods are often laborious and time-intensive, and thus an alternative high-throughput approach is needed.

Genetics, and in particular genetic heritability, can be used as an unbiased and high-throughput metric to quantitatively benchmark and compare different phenotyping approaches, because nearly all human traits, including dietary intake, are influenced by genetic variation, either directly or indirectly (Ge et al., 2017a; Cole et al., 2020). This ubiquity of an underlying biological association allows our approach to use a common multi-variable human reference (i.e., the human genome) to estimate a summary aggregate variable of association (i.e., heritability) rather than rely on phenotype-specific gold-standard correlates. Furthermore, unlike other biological -omics datasets, genotypes also benefit from being captured in an unbiased and accurate manner nearly evenly throughout the genome, their easy accessibility, their increasing affordability, and their stability through an individual’s lifetime with their consequent robustness to environmental confounders.

In this brief report we outline a preliminary investigation of genetic heritability as an anchor to compare relative validity of the same phenotypes derived using different statistical transformations, and we test its use at scale on hundreds of longitudinal 24HR questionnaire dietary variables from approximately 176 K individuals in UK Biobank (UKB). A flow chart of the study design is included in Supplementary Figure S1. Specifically, we derive a set of EB food intake variables over multiple 24HR questionnaires per person and compare these EB weighted values to crude unweighted estimates of either how often the food or beverage was consumed (proportions: Number of times consumed/number of questionnaires taken) or how much was consumed (averages: average amount over multiple questionnaires) using heritability analysis. Ultimately, we use heritability as a proxy for phenotype quality to determine if and when the EB method outperforms its crude counterpart across multiple variables simultaneously without the need for known gold standard correlates.

## Methods

### UK Biobank sample

UK Biobank is a prospective cohort of 500 K adults ages 40–69 at baseline collected from 2006 to 2010 across the UK. This large biomedical and research resource contains biological samples used to derive genetics, metabolomics, proteomics, and biomarkers as well as detailed phenotyping information spanning physical measures, imaging, lifestyle questionnaires, and health outcomes from multiple sources (self-reported, nurse interviews, and linked medical records). Extensive details on the genotyping, imputation, and quality control of this data, in addition to methodological details on deriving a subset of individuals of European ancestry (*N* = 455,146) used herein have been described elsewhere (Bycroft et al., 2018; Cole et al., 2020). All individual-level analyses were conducted under UKB application 11898 in compliance with UKB regulations and all participants provided informed consent.

### Dietary phenotype derivation

UKB contains data from two distinct dietary intake questionnaires. The first is a brief modified food frequency questionnaire (FFQ) of roughly 30 questions pertaining to habitual intake and frequency of foods and beverages over the previous year, asked of all participants in-person using a touchscreen at their baseline assessment center visit. The second, which is the sole dietary data source for this study, is a detailed 24HR questionnaire in which a subset of participants answered over 200 questions on specific foods and beverages consumed (with quantities) in the preceding 24-hour day. The 24HR was implemented as a questionnaire for the final 70 K in-person baseline assessment center participants from 2009–2010 and emailed four times to 320 K participants who consented to re-contact *via* email between February 2011 and April 2012. Approximately 200 K individuals have at least one and up to five recorded 24HR questionnaires.

Each questionnaire was filtered for credible estimates of total energy intake [≥1,000 kJ (UKB field 100002) and ≤20 MJ for males and ≤18 MJ for females (UKB field 100026)], typical dietary intake (UKB fields 100020 and 20085), completion duration greater than or equal to 5 min (UKB field 20082), and overall completion (UKB field 20081). Additionally, the participant could not be pregnant within 1 year of taking the 24HR nor have a cancer diagnosis within the previous year (UKB fields 3,140 and 40005). All 24HR questions were converted into 1/0 for yes/no to consumption; each categorical response was coded similarly [e.g., UKB field 20086 for special diet was converted into six binary variables for each response (gluten-free, lactose-free, low calorie, vegetarian, vegan, and a combined vegetarian or vegan field)]. 24HR questions pertaining to quantity consumed were also included as continuous variables.

After individual questionnaire pre-processing, all available data from all questionnaires were combined into two types of phenotypes: “proportions” for all food items representing the number of times consumed over the total number of questionnaires taken, and “averages” of continuous items only (i.e., quantities) which are simply averages over multiple questionnaires taken. Each phenotype type (proportions and averages) was derived using two approaches for comparison: “crude,” representing the simple un-weighted derivations as indicated above, and “Empirical Bayes (EB),” which applies the EB method to weight individual responses based on the number of questionnaires taken.

EB proportions were calculated by first estimating empirical distribution parameters (alpha and beta) from a zero-one inflated distribution fit using the gamlssInf0to1 function in the gamlss.inf and gamlss R packages (Stasinopoulos et al., 2017), then calculating an EB proportion as follows: (number of successes + alpha)/(total number of questionnaires + alpha + beta). Of note, two nearly homogenous variables did not converge (UKB field 100920 milk type: “any” and a combined total drinks variable); for these we used parameters estimated from a similar variable distribution (UKB field 100920 any dairy milk type: “semiskimmed,” “skimmed,” and/or “whole”). EB averages were calculated by first fitting a Dirichlet-multinomial mixture model to all continuous variables as a matrix of possible responses and counts for each response as implemented in the DirichletMultinomial R package (Morgan, 2022) This fit model empirically estimates an alpha parameter to update each individual response based on the raw values and counts. Once a weighted value is obtained for each possible response, each individual’s EB average is obtained by summing their weighted values over the total number of questionnaires plus the sum of the alpha estimates. A detailed explanation with R code has been described previously (Robinson, 2017).

### Using genetics to benchmark phenotype precision

To estimate heritability, we first conducted genome-wide association study (GWAS) analysis on each phenotype using REGENIE whole genome regression software (version1.0.6.7), which allows for the inclusion of related individuals using a model similar to a linear mixed model (Mbatchou et al., 2021). Briefly, we first prepared a set of quality-controlled markers by filtering to genotyped markers with minor allele frequency >0.5%, minor allele count >10, and missingness <10% in samples of genetically determined European ancestry (see above) with less than 10% genotype missingness (M = 784,256). We next conducted REGENIE as directed in two steps, first fitting a whole genome regression model capturing the phenotypic variance attributable to genetic effects, followed by testing the association between each 24HR diet phenotype and 58,299,817 imputed markers conditional upon the model in step one. The resulting genetic variants were filtered for imputation INFO score ≥0.6 and common variants with minor allele frequency ≥0.5%, resulting in genome-wide summary statistics for 11,006,968 variants across 1,288 total phenotypes.

Our specific analysis presented here was computationally intense and required a high-performance computing environment. REGENIE linear mixed model GWAS for 1,288 phenotypes in ∼200 K individuals required splitting the data into six sets, each requiring approximately 30 GB of memory and 10 days of compute time. For more information on performance, please see the REGENIE documentation (https://rgcgithub.github.io/regenie/). Several factors would improve the computational cost of this approach including more heritable phenotypes in smaller sample sizes and the use of traditional (and not mixed) linear or logistic GWAS models.

The following covariates were included in the genetic model: sex, average age in months, average age in months squared, assessment center (UKB field 54 as a factor), birthplace (UKB field 1,647 as a factor), self-reported ethnicity (UKB field 21000 as a factor), proportion of questionnaires taken on a weekend (Friday, Saturday, or Sunday; UKB field 20080), duration of questionnaire in minutes winsorized at 25 min (UKB field 20082), the proportion of questionnaires taken with a duration ≥25 min (UKB field 20082), average hour of the day completed (UKB field 20081), total number of questionnaires taken, ten genetic principal components derived previously (Cole et al., 2020), and genotyping array. We used LD score regression software (version1.0.0) and LD scores computed using 1,000 Genomes European data to extract heritability estimates of each 24HR dietary phenotype. A Bonferroni heritability significance threshold was obtained by dividing 0.05 by the number of effectively independent phenotypes (*N* = 46.1) as estimated by the “eigenvalue formula” (Bretherton et al., 1999) on eigenvalues obtained from a principal components analysis on all covariate-adjusted dietary variables.

Intra-class correlation, a measurement of consistency across measures (e.g., across multiple 24HR questionnaires), was calculated as the between-subject variance/(between-subject variance + within-subject variance) from individuals of European ancestry that took the questionnaire all 5 times (*N* = 2,066) in R with the “irr” package. The clump command within the PLINK2 software (Chang et al., 2015) and the 1000 Genomes Project phase 3 European reference (Auton et al., 2015) was used to determine the number of independent genome-wide significant loci (*P* < 5 × 10^−8^) in 500 kb windows in each GWAS, followed by collapsing signals across all GWAS together to leave only one lead SNP-phenotype association per window.

## Results

The 24HR questionnaire contains over 200 questions on foods and beverages consumed in the previous 24-hour day. After individual 24HR questionnaire quality control and filtering, there were 176,858 individuals remaining for all downstream analysis. Among these individuals, over half took the questionnaire at least twice (*N* = 95,777; 54%) with 46,893 completing two, 31,818 completing three, 15,000 completing four, and 2,066 completing all five 24HR questionnaires (Supplementary Figure S2).

**FIGURE 1:**
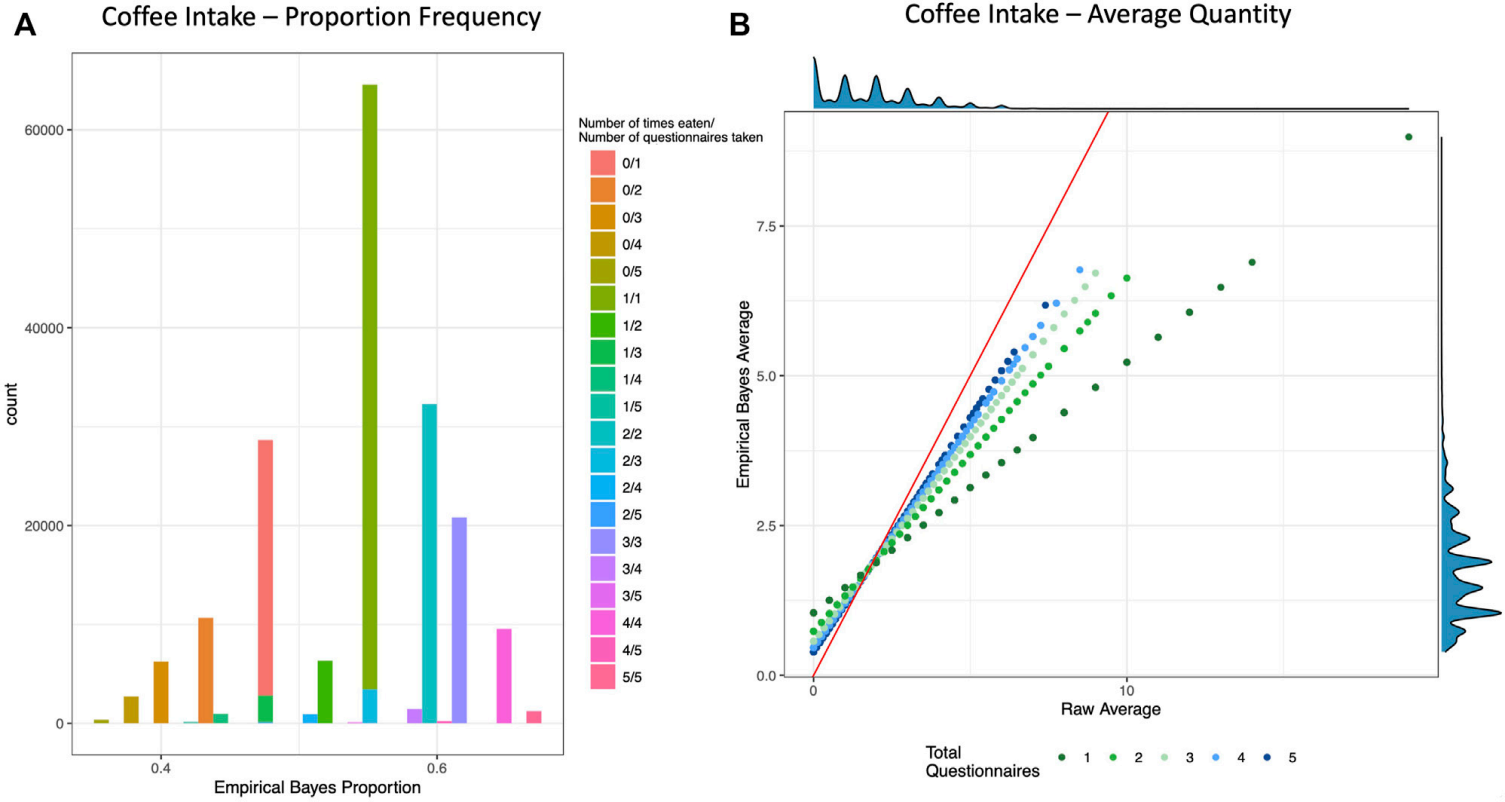
Empirical Bayes Proportion and Average Transformations: Coffee Intake. These example visualizations of coffee intake depict the shrinking of raw values for individuals with fewer total questionnaires. **(A)** Histogram of Empirical Bayes proportion colored and stacked by the crude proportion (number of times an individual reported drinking coffee out of how many total questionnaires that individual took). **(B)** Scatter plot of the crude average (*x*-axis) vs. the Empirical Bayes Average (*y*-axis) of cups of coffee per day, colored by total number of questionnaires taken. The red line is the line of identity, and the density plots are depicted on the top and right borders. See Supplementary Figure S3 for more examples.

From 264 UKB 24HR questionnaire fields, many with multiple categorical responses, we derived 158 binary variables (yes/no to consumption) and 243 continuous variables (quantities). All variables were converted to proportions (how often a food/beverage was consumed over questionnaires taken) and all continuous quantities were also averaged over questionnaires taken. Finally, all variables underwent an EB transformation as described in the *Methods* section, resulting in both a crude and EB version for each phenotype, for a total of 1,288 phenotypes tested for downstream analysis (Supplementary Table S1; Figure 1; Supplementary Figure S3). Note, averages were calculated from all questionnaires taken for each individual, even when that food or beverage was not consumed (i.e., a quantity of 0). Therefore, the accuracy of average quantities of foods and beverages that are episodically consumed on an irregular basis will likely be lower than the accuracy of average quantities of foods and beverages that are more regularly consumed. An alternative averaging approach, which was not taken in this study, would be to average quantities of foods only from questionnaires in which the food was consumed or apply more sophisticated approaches for episodic foods as previously developed (Kipnis et al., 2009).

**FIGURE 2:**
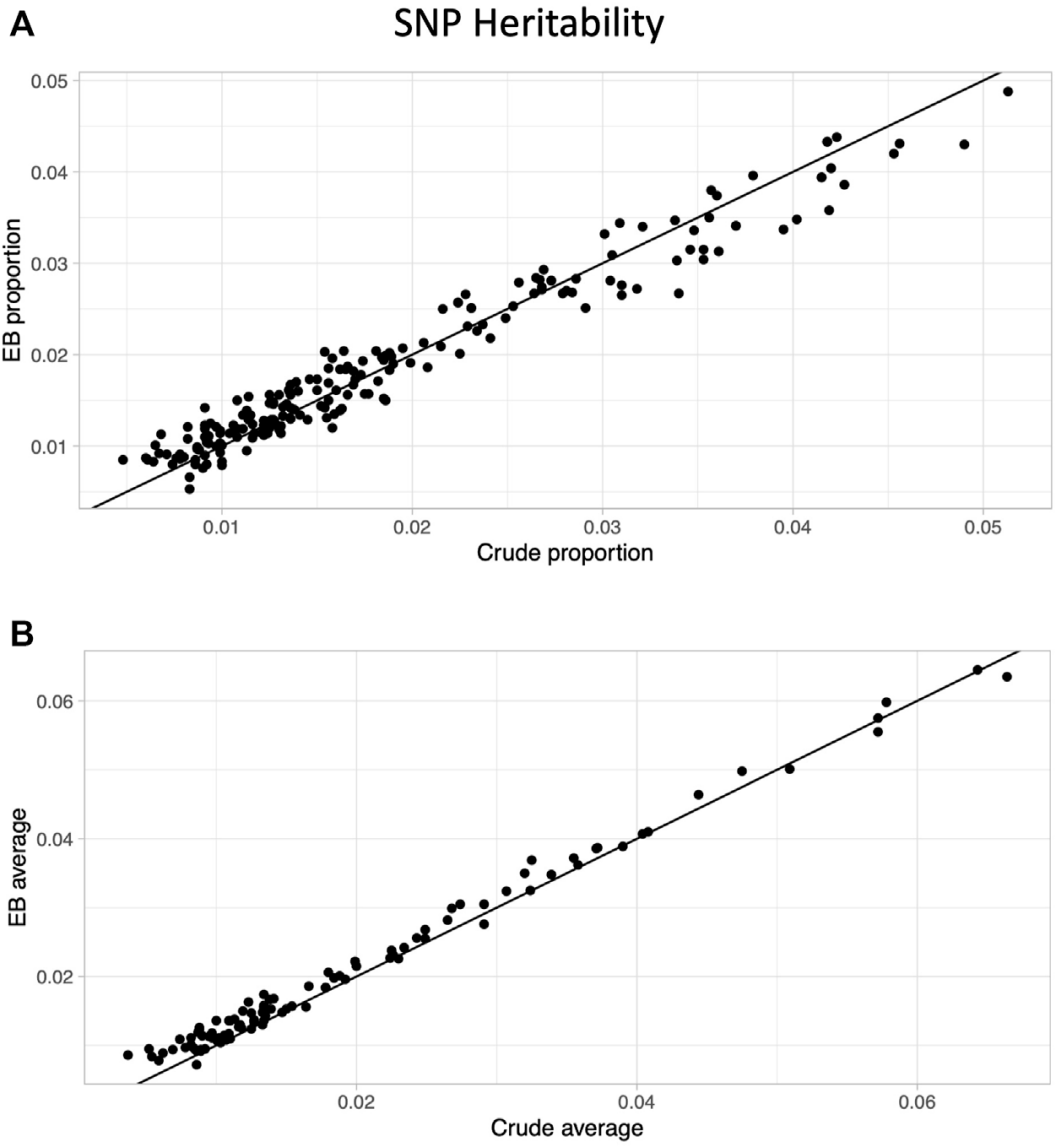
Heritability comparison between crude and Empirical Bayes approaches. Scatter plots of SNP heritability estimates comparing crude (*x*-axis) and Empirical Bayes (*y*-axis) for proportion phenotypes **(A)** and average phenotypes **(B)**. The black line is the line of identity.

After limiting to phenotypes in which at least one approach (crude or EB) had a significant heritability estimate based on a multiple testing threshold corrected for effectively independent phenotypes (*p* < .05/46.1 = 0.00108; see *Methods*), 200 proportion and 102 average quantity phenotypes remained. The EB approach led to higher heritability for well over half the phenotypes (209/302 = 69%), and the improvement in heritability was much more prominent in the average quantity (91/102 = 89%) compared with the proportion phenotypes (118/200 = 59%; Figure 2).

**FIGURE 3:**
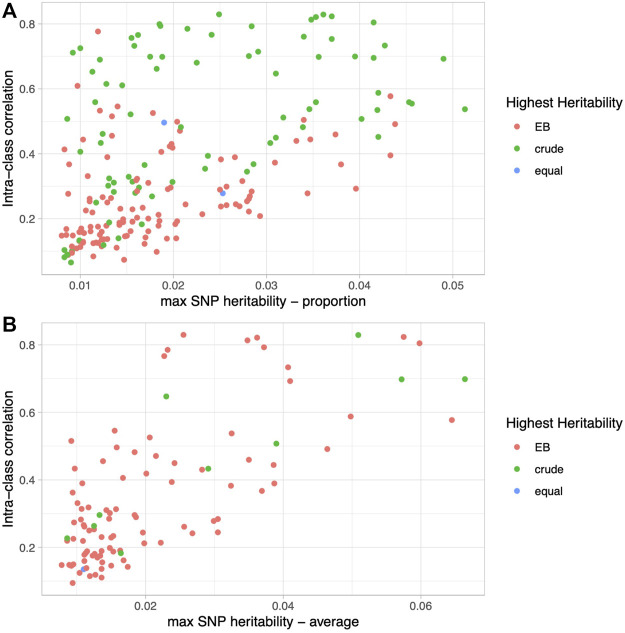
Relationship between phenotype reliability and heritability. Scatter plots of intra-class correlation (*y*-axis) versus SNP heritability estimates (*x*-axis) colored by the method (EB, crude, or equal) that led to the higher heritability for proportion phenotypes **(A)** and average phenotypes **(B)**.

Upon closer examination of the dietary proportion phenotypes, we noticed that the EB approach led to higher heritability estimates at the lower end of the heritability spectrum, while the crude proportions led to higher heritability estimates at the higher end of the spectrum. We hypothesized that foods and beverages that are consumed on a more regular basis and have less questionnaire-to-questionnaire variability would have the highest heritability estimates and benefit the least from our version of the EB approach. To test this, we calculated intra-class correlation, a measure of reliability across multiple measures, on all raw dietary variables from a subset of individuals that took all five 24HR questionnaires (*N* = 2,066; Supplementary Table S1). Not surprisingly, there is a strong correlation between the ICC (i.e., the reliability from questionnaire to questionnaire) and the estimated crude SNP heritability (overall correlation = 0.61, proportions = 0.54, averages = 0.74; Figure 3), with the highest heritability among the most reliable phenotypes.

Furthermore, as seen in Figure 3A and Supplementary Table S1, the crude approach consistently leads to higher heritability estimates than the EB approach among the most reliable phenotypes such as coffee intake, and *vice versa* among the least reliable phenotypes, such as chocolate intake. Specifically, for the proportions with ICC in the top quartile (ICC ≥ 0.513), the crude proportion leads to higher heritability 86% of the time (43/50 phenotypes), whereas for those derived from the least reliably reported foods and beverages in the bottom quartile (ICC ≤ 0.178), the EB proportion leads to higher heritability 84% of the time (42/50 phenotype comparisons). On the other hand, average quantities of foods and beverages, whether reliably reported from questionnaire to questionnaire or not, have consistently higher heritability estimates using the EB approach: 91% (21/26) in the top quartile (ICC ≥ 0.458) and 92% (24/26) in the bottom quartile (ICC ≤ 0.188) (Figure 3B). Habitually consumed beverages (e.g., coffee, water, tea, and alcohol) are among the most reliable (i.e., high ICC) and heritable phenotypes, and demonstrate this phenomenon well (Supplementary Figure S4). Although crude proportions of habitually consumed beverages have higher heritabilities, the EB version leads to higher heritability among the average quantity phenotypes, and even more so when the ICC is low.

Although gold standards are typically not available for most dietary phenotypes, some dietary phenotypes have strong associations at genetic loci with well-established mechanisms, which can serve as “genetic gold standards” for this subset of phenotypes. More broadly, if heritability were an appropriate metric to confidently assign and rank phenotype quality among different processing approaches, we would expect the more heritable version to have a stronger statistical association at genetic loci, particularly those with established biological mechanisms. To evaluate this question, we investigated the top associations from our GWAS data. Overall, we find that 208/379 (55%) of our independent loci associated with dietary intake (See *Methods*) are more strongly associated with the more heritable phenotype version (164 crude and 214 EB). Notably, these loci include well-known genetic gold standard associations such as SNP rs2472297 near the *CYP1A2* caffeine metabolism gene associated with coffee intake (Faber et al., 2005) and SNP rs2708381 in the *TAS2R46* bitter taste receptor gene (Andres-Barquin and Conte, 2004) associated with adding sugar or artificial sweetener to different beverages and foods. When filtering to dietary traits with the largest percent difference in heritability between the two versions (top 25% and top 10%), this concordance increases to 67% and 77%, respectively. This suggests that heritability may need to be substantially different to increase GWAS association strength.

## Discussion

The overall goal of our study was to apply an EB approach to account for variability in number of repeated measures in dietary data and use an unbiased metric for assessing its utility in a high-throughput manner. While gold standard measurements are often used in epidemiology to assess validity, they are often limited, unknown, or unmeasured in practice. Heritability provides a simple and broadly applicable extension of this approach that capitalizes on the measurable, non-zero heritability of the great majority of phenotypes (Ge et al., 2017a), meaning that a portion of their phenotypic variance is explained by genetic variance. Even if this heritability derives from a different, heritable mediator phenotype (as is often the case with largely environmentally-driven traits like dietary intake), increased precision in phenotypic measurement will result in reduced observed phenotypic variance and hence increased estimated heritability. Here, we use heritability estimates as an unbiased metric to compare the relative validity of phenotype processing approaches, and apply this standard simultaneously across hundreds of dietary variables.

Unlike the dietary data in UKB, typical nutritional epidemiology-focused cohorts capture dietary intake more often, at regularly spaced intervals, and validate with multiple different questionnaires (WILLETT et al., 1985; Ocké et al., 1997). Still, previous work has found that dietary variables derived from the 24HR questionnaire in UKB have ICC and correlations with biomarkers comparable to those derived from the more burdensome conventional studies (Carter et al., 2019; Greenwood et al., 2019). Furthermore, although dietary intake is a behavioral trait that is influenced by many external health and socio-cultural factors, we find that 302 of our overlapping derived dietary phenotypes have significant, albeit modest, heritabilities. Together, these findings support the utility of the UKB 24HR questionnaire data for capturing meaningful information for future studies, potentially in combination with the UKB FFQ, which alone does not contain enough information to estimate energy and nutrient intake.

We apply a Bayesian approach using the empirical data at hand to estimate distribution parameters and update individual estimates of proportion and average quantity phenotypes, representing how often and how much a food or beverage is consumed, respectively. The EB approach leads to higher heritability estimates more often than its crude counterpart, most often when considering average quantities consumed, and least often when examining yes/no questionnaire variables for foods and beverages that are consumed habitually with high reproducibility. There is a wide array of research and literature on accounting for measurement error in 24HR questionnaires, and future work could expand upon this brief report to compare these additional approaches to each other under different circumstances, such as among different dietary intake classes (e.g., foods, food groups, nutrients, and dietary patterns) or underlying frequency (e.g., habitual and episodic) (Kipnis et al., 2009; Bennett et al., 2017). We speculate, based on the findings within, that the more stable and reliable the dietary trait, such as with macronutrient levels, the less of a noise reduction and power gain would be seen using the Empirical Bayes and other measurement error correction methods.

In summary, we provide support for using heritability estimates as a novel tool for assessing phenotype quality in a high throughput manner, leveraging relationships with genetic variation on thousands of individuals as a common reference for hundreds of traits. A key feature that makes this type of analysis a viable and scalable approach is the stable and consistent genetic backbone that all individuals share, which genome-wide genotyping data are making more readily available in many large cohorts and biobanks throughout the world. Together with a thoughtful understanding of the biological question at hand, heritability can be used to optimize dietary variable processing and phenotype derivation. This approach can be extended to many traits and phenotype processing approaches beyond the field of nutritional epidemiology, as the principle of this work only hinges on a non-zero heritability. However, a key limitation to this approach is that heritability must be detectable. We demonstrate that the large sample size of the UKB allowed us to detect even modest heritability for many but not all noisy and environmentally mediated dietary traits derived from UKB’s 24HR questionnaire. Furthermore, unlike correlations with known biomarkers, our use of heritability only quantifies the relative precision of dietary phenotypes, and does not discern their accuracy, particularly if mediated (and to different extents) through another heritable trait, as is often the case with dietary intake (e.g., health conditions and socioeconomic status) (Pirastu et al., 2022). Complete mediation of the relationship between genetic variants and dietary intake by heritable health conditions (e.g., medical advice that changes eating habits) would limit the use of this approach in a population free of the condition at hand. In the end, heritability is a metric of an underlying biological relationship, direct or indirect, with the phenotypes at hand; therefore, a key assumption when comparing the same phenotype processed in two different ways is that the same genetic variants are at play, and the heritability estimate is capturing phenotype precision alone. As discussed, the use of heritability as a precision metric is best suited for comparing different transformations of the same phenotype, but an important next question is then how to compare heritability between two different phenotypes with both different levels of phenotype precision *and* different underlying genetic determinants. Furthermore, applying a recently developed approach that estimates heritability after correcting for measurement error (Ge et al., 2017b) to the nutritional data in UKB is a compelling and complementary next step to truly determine which dietary traits are more heritable.

## Data Availability

The original contributions presented in the study are included in the article/Supplementary Material, further inquiries can be directed to the corresponding author.
