# Corrigendum: Genetic heritability as a tool to evaluate the precision of 24-hour recall dietary questionnaire variables in UK Biobank

**DOI:** 10.3389/fgene.2023.1202158

**Published:** 2023-05-04

**Authors:** Joanne B. Cole, Kenneth E. Westerman, Alisa K. Manning, Jose C. Florez, Joel N. Hirschhorn

**Affiliations:** ^1^ Programs in Metabolism and Medical and Population Genetics, The Broad Institute of MIT and Harvard, Cambridge, MA, United States; ^2^ Diabetes Unit and Center for Genomic Medicine, Massachusetts General Hospital, Boston, MA, United States; ^3^ Department of Medicine, Harvard Medical School, Boston, MA, United States; ^4^ Division of Endocrinology, Boston Children’s Hospital, Boston, MA, United States; ^5^ Department of Biomedical Informatics, University of Colorado School of Medicine, Aurora, CO, United States; ^6^ Clinical and Translational Epidemiology Unit, Mongan Institute, Massachusetts General Hospital, Boston, MA, United States; ^7^ Department of Genetics, Harvard Medical School, Boston, MA, United States

**Keywords:** heritability, nutrigenomics, nutritional epidemiology, 24-hour diet recall questionnaires, relative validity, phenotyping, empirical bayes, longitudinal data

In the published article, there was a coding error that led to an incorrect finding in the **Results**. The updated **Result** continues to emphasize both the potential of this novel approach and the need for future investigation.

Specifically, a correction has been made to the **Results** Section, paragraph 6. The previous version stated:

“Although gold standards are typically not available for most dietary phenotypes, some dietary phenotypes have strong associations at genetic loci with well-established mechanisms, which can serve as “genetic gold standards” for this subset of phenotypes. More broadly, if heritability were an appropriate metric to confidently assign and rank phenotype quality among different processing approaches, we would expect the more heritable version to have a stronger statistical association at genetic loci, particularly those with established biological mechanisms. To evaluate this question, we investigated the top associations from our GWAS data. Overall, we find that all 195 of our independent loci associated with dietary intake (See **Methods**) are more strongly associated with the more heritable phenotype version (54 crude and 141 EB). Notably, these loci include well-known genetic gold standard associations such as SNP rs1229984 in the ADH1B alcohol dehydrogenase gene associated with alcohol intake (Peng and Yin, 2009), SNP rs2472297 near the CYP1A2 caffeine metabolism gene associated with coffee intake (Faber et al., 2005), and SNP rs2708381 in the TAS2R46 bitter taste receptor gene (Andres-Barquin and Conte, 2004) associated with adding sugar or artificial sweetener to different beverages and foods.”

The corrected section appears below:

“Although gold standards are typically not available for most dietary phenotypes, some dietary phenotypes have strong associations at genetic loci with well-established mechanisms, which can serve as “genetic gold standards” for this subset of phenotypes. More broadly, if heritability were an appropriate metric to confidently assign and rank phenotype quality among different processing approaches, we would expect the more heritable version to have a stronger statistical association at genetic loci, particularly those with established biological mechanisms. To evaluate this question, we investigated the top associations from our GWAS data. Overall, we find that 208/379 (55%) of our independent loci associated with dietary intake (See **Methods**) are more strongly associated with the more heritable phenotype version (164 crude and 214 EB). Notably, these loci include well-known genetic gold standard associations such as SNP rs2472297 near the CYP1A2 caffeine metabolism gene associated with coffee intake (Faber et al., 2005) and SNP rs2708381 in the TAS2R46 bitter taste receptor gene (Andres-Barquin and Conte, 2004) associated with adding sugar or artificial sweetener to different beverages and foods. When filtering to dietary traits with the largest percent difference in heritability between the two versions (top 25% and top 10%), this concordance increases to 67% and 77%, respectively. This suggests that heritability may need to be substantially different to increase GWAS association strength.”

The authors apologize for this error and state that this does not change the scientific conclusions. The original article has been updated.

