# Supplementary material for: Genetic heritability as a tool to evaluate the precision of 24-hour recall dietary questionnaire variables in UK Biobank
Source: Front Genet. 2023 Jan 4;13:1070511. doi: 10.3389/fgene.2022.1070511 (PMC9845390; doi:10.3389/fgene.2022.1070511)

frontiers

Supplementary Material

Genetic heritability as a tool to evaluate the precision of dietary variables derived from longitudinal 24-hour recall questionnaires in UK Biobank

Joanne B. Cole[1-5,*], Kenneth E. Westerman[1,3,6], Alisa K. Manning[1,3,6], Jose C. Florez[1-3], Joel N. Hirschhorn[1,4,7]

[1] Programs in Metabolism and Medical and Population Genetics, The Broad Institute of MIT and Harvard, Cambridge, MA, USA

[2] Diabetes Unit and Center for Genomic Medicine, Massachusetts General Hospital, Boston, MA, USA
[3] Department of Medicine, Harvard Medical School, Boston, MA, USA
[4] Division of Endocrinology, Boston Children's Hospital, Boston, MA, USA

[5] Department of Biomedical Informatics, University of Colorado School of Medicine, Aurora, CO, USA

[6] Clinical and Translational Epidemiology Unit, Mongan Institute, Massachusetts General Hospital, Boston, MA, USA

[7] Department of Genetics, Harvard Medical School, Boston, MA, USA

* Corresponding Author: Joanne B. Cole; joanne.cole@cuanschutz.edu

Supplemental Figure 1: Study design flowchart.

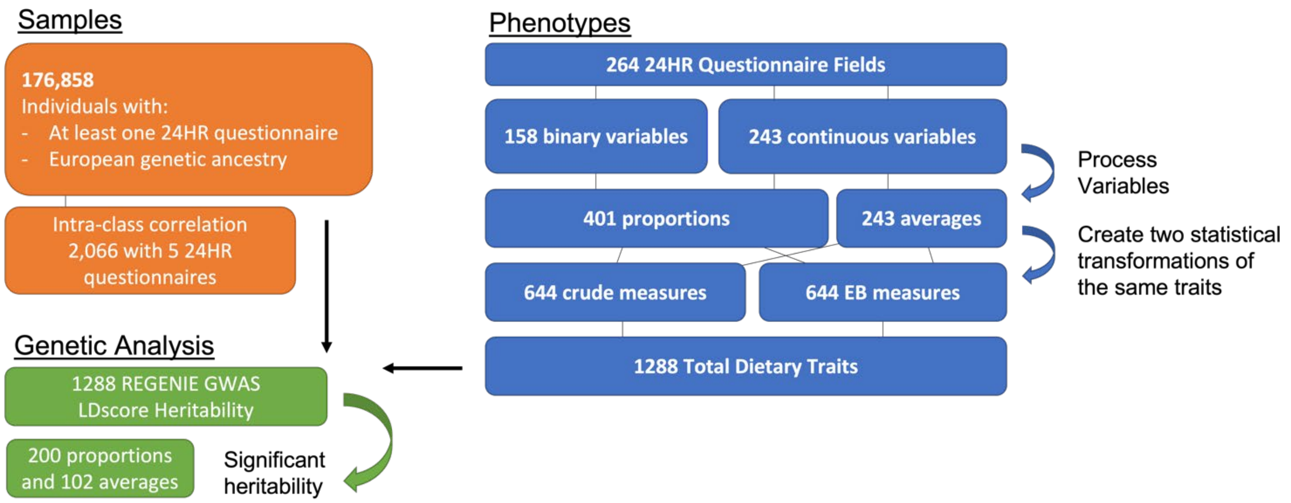

Supplemental Figure 2: Histogram of total number of 24-hour recall questionnaires taken in the final study sample.

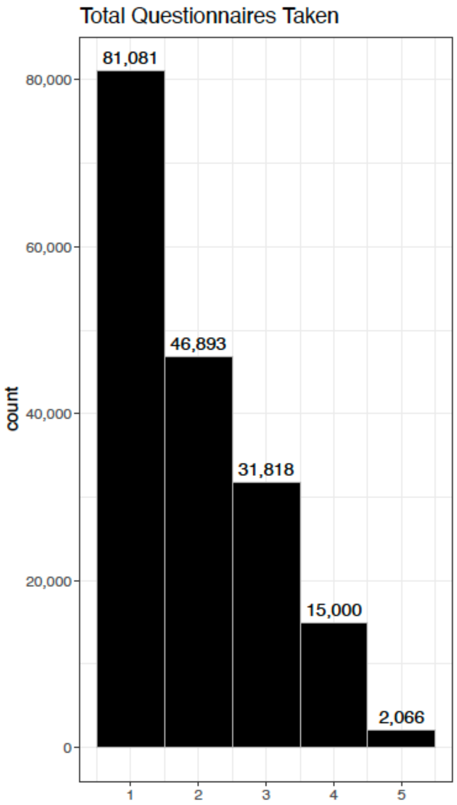

Supplemental Figure 3: Empirical Bayes Proportion and Average Transformations: Meat, Mushroom, and Nut Intake. These example visualizations depict the shrinking of raw values for individuals with fewer total questionnaires. A) Histogram of Empirical Bayes proportion colored and stacked by the crude proportion (number of times an individual reported consumption out of how many total questionnaires that individual took). B) Scatter plot of the crude average (x-axis) vs. the Empirical Bayes Average (y-axis), colored by total number of questionnaires taken. The red line is the line of identity, and the density plots are depicted on the top and right borders.

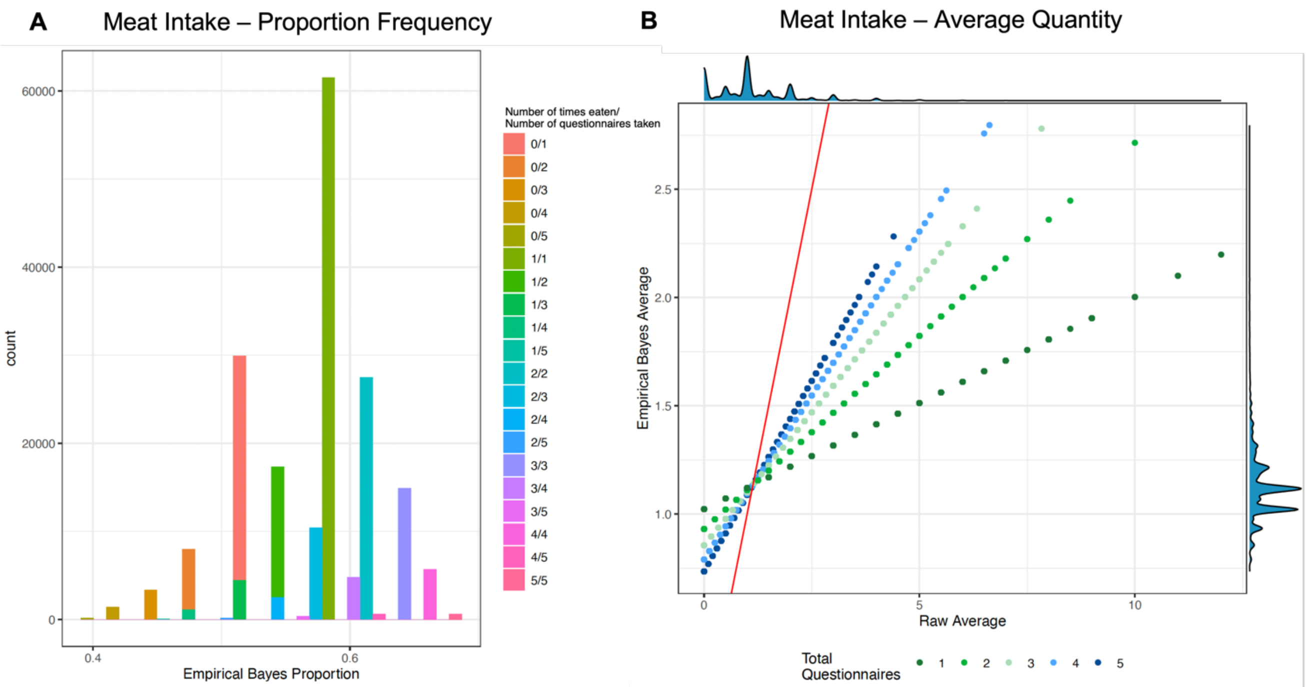

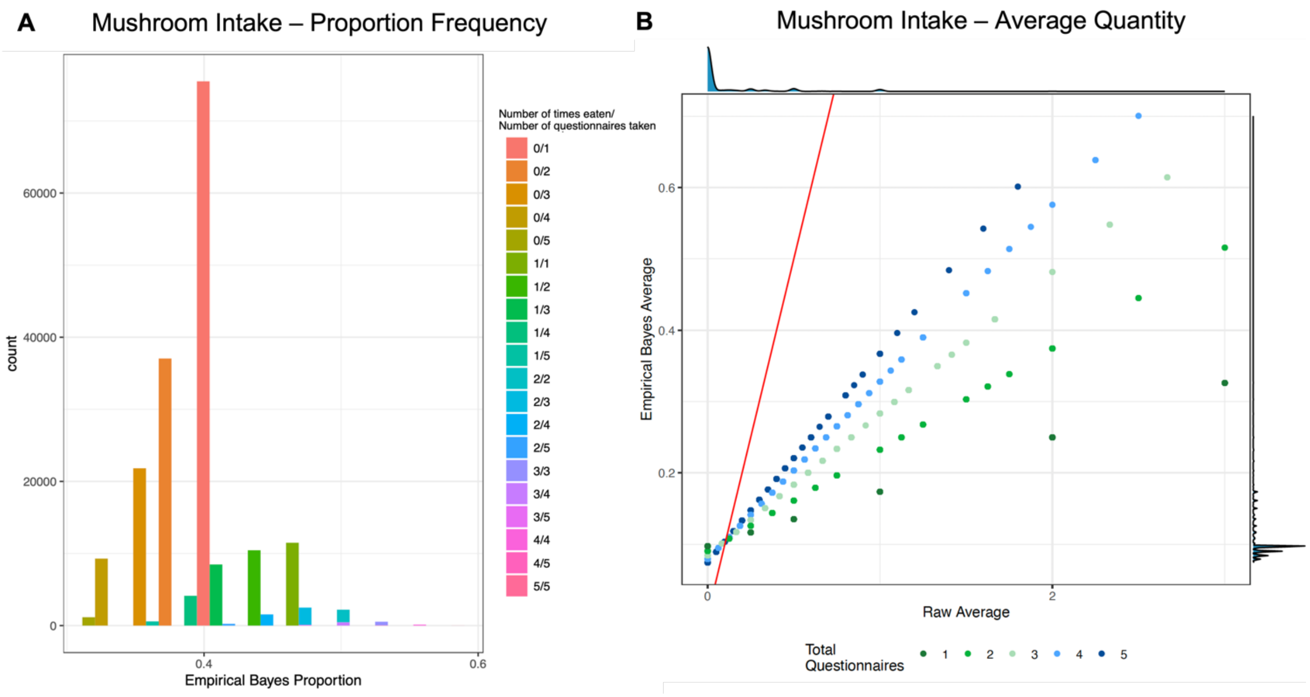
A
Mushroom Intake – Proportion Frequency
count
Empirical Bayes Proportion
Number of times eaten/
Number of questionnaires taken
0/1
0/2
0/3
0/4
0/5
1/1
1/2
1/3
1/4
1/5
2/2
2/3
2/4
2/5
3/3
3/4
3/5
4/4
4/5
5/5
B
Mushroom Intake – Average Quantity
Empirical Bayes Average
Raw Average
Total
Questionnaires
1
2
3
4
5

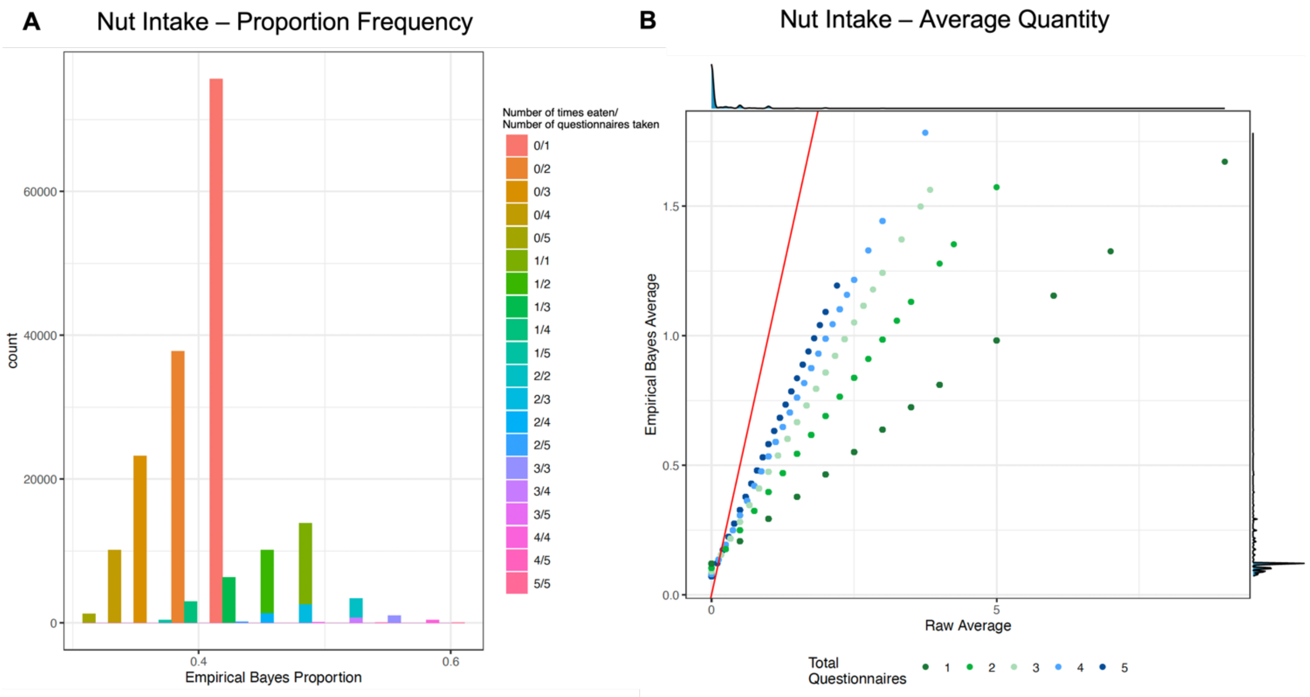
A
Nut Intake – Proportion Frequency
count
Empirical Bayes Proportion
Number of times eaten/
Number of questionnaires taken
0/1
0/2
0/3
0/4
0/5
1/1
1/2
1/3
1/4
1/5
2/2
2/3
2/4
2/5
3/3
3/4
3/5
4/4
4/5
5/5
B
Nut Intake – Average Quantity
Empirical Bayes Average
Raw Average
Total
Questionnaires
1
2
3
4
5

Supplemental Figure 4: A replica of Figure 3 with all beverage phenotypes displayed as triangles with black borders.

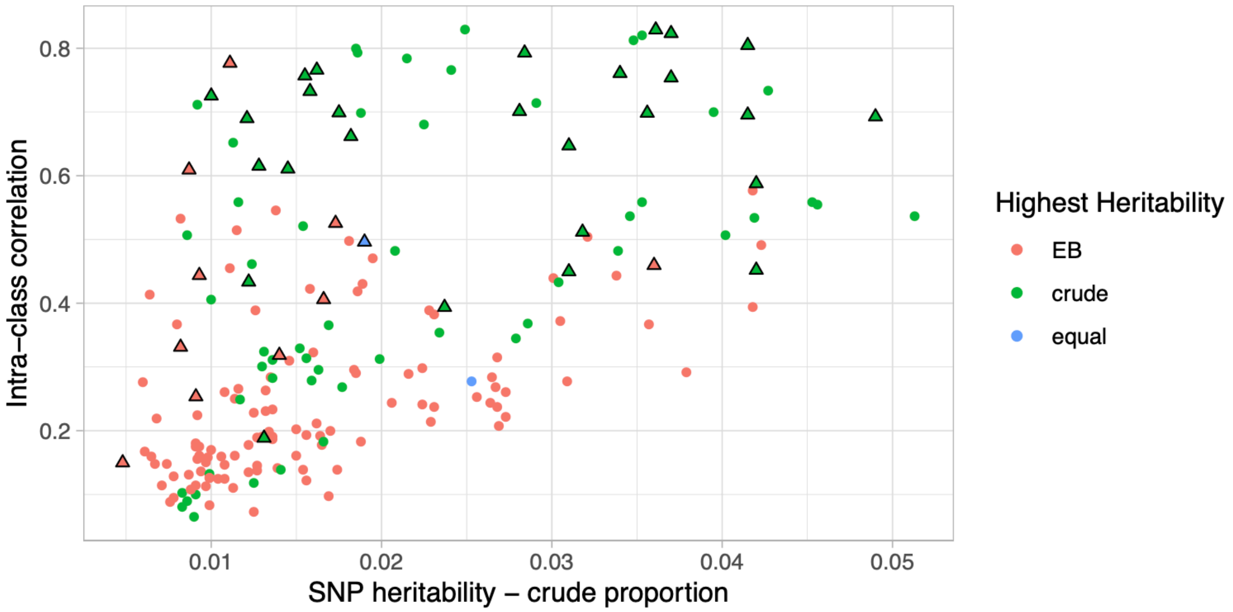

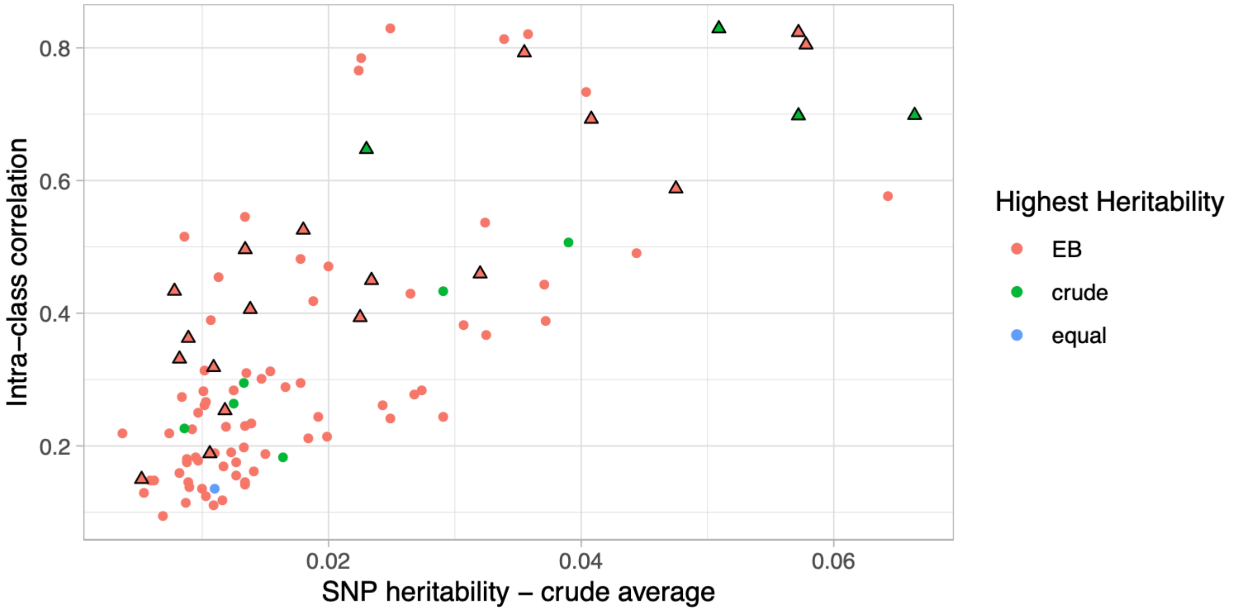

Supplement: Supplementary file 2 [file Image1.pdf]
